# Prognostic factors for disease progression of central high-grade conventional osteosarcoma of the appendicular skeleton: Single-centre experience within South Africa with minimum 3-year follow-up

**DOI:** 10.1016/j.jor.2024.12.019

**Published:** 2024-12-25

**Authors:** PhakamaniG. Mthethwa, L.C. Marais

**Affiliations:** a Head of Department of Orthopaedic Surgery, Consultant of Bone Tumours, Sepsis, and Limb Reconstruction, Dr Pixley Ka Isaka Seme Memorial Hospital, University of KwaZulu-Natal – Nelson Mandela School of Clinical Medicine, 310 Bhejane Street, KwaMashu, 4360, Durban, South Africa; b Head of School, Sepsis, and Limb Reconstruction, Nelson Mandela School of Clinical Medicine, College of Health Sciences, University of KwaZulu-Natal, 719 Umbilo Road, 4001, Durban, South Africa

**Keywords:** Disease progression, High-grade conventional osteosarcoma, Relapse, Extremities, Low- and middle-income countries

## Abstract

**Background:**

Disease progression (DP) of osteosarcomas, albeit with aggressive treatments, hinders improving survival. The DP patterns are unique in low- and middle-income countries like South Africa. We determine the prognostic factors associated with disease progression (DP) of the appendicular skeleton's central high-grade conventional osteosarcoma (COS).

**Methods:**

This is a retrospective study of 77 patients, with a minimum 3-year follow-up period diagnosed with histological biopsy-confirmed COS. Descriptive statistics, Cox proportional regression modelling, and the Kaplan-Meier method were employed for the analysis.

**Results:**

DP occurred in 75 % of patients (58/77), either as a local progression - LP 32 % (25/77), systemic progression – SP 61 % (47/77) or both 32 % (24/77). In the univariate analysis, the factors associated with DP were proximal humerus tumor site (hazard ratio [HR] 2.48; 95 % confidence interval [CI], 1.02 to 6.04; p < 0.046), metastasis at diagnosis (HR 1.91; 95 % CI, 1.10 to 3.32; p < 0.022), multiple metastatic lesions (HR 2.58; 95 % CI, 1.13 to 5.88; p < 0.024), curative treatment (HR 0.33; 95 % CI 0.17 to 0.62; p < 0.001), palliative treatment (HR 2.17; 95 % CI 1.24 to 3.78; p < 0.007), and wide surgical resection (HR 0.48, 95 % CI 0.27 to 0.86; p < 0.013). On multivariate analysis, only age >19 years was an independent risk factor (HR 1.04; 95 % CI 1.00 to 1.08; p < 0.034). The median survival time was 24 months, with an overall survival (OS) of 57.1 % at 3 years. The projected Kaplan- Meier 5-year OS rate was 29.78 %, with a progression-free survival (PFS) rate of 10.28 % (HR 0.76; 95 % CI 0.52 to 1.112; p < 0.128).

**Conclusion:**

In this series of central high-grade conventional osteosarcoma of the appendicular skeleton from South Africa, we observed a uniquely high proportion of disease progression (DP). Age >19, metastatic disease, and no chemotherapy response yielded poor outcomes; in contrast, wide surgical resection is beneficial. Further elucidation is needed at a larger scale in this region.

**Study evidence level:**

IV.

## 1 Introduction

Osteosarcomas are a group of aggressive primary malignant bone tumours of mesenchymal cancer stem cells origin that produce an osteoid matrix with predilection in childhood and young adults.^1^ Central high-grade conventional osteosarcoma (COS) is the most common subtype, comprising 90 % of all variants.^1^^,^^2^ Despite advances in multidrug chemotherapy and surgical procedures, the 3-year event-free survival (EFS) and 5-year overall survival (OS) of nonmetastatic COS have plateaued at 60–70 %.^3^, ^4^, ^5^, ^6^, ^7^ In contrast, previous African studies have reported poor survival rates, with 5-year overall survival rates ranging from 30 to 57 %.^8-11.^ It remains unclear whether this decrease in survival compared to that in the developed world results from genetic variation or treatment-related factors.

Tumour response to chemotherapy has emerged as the most important prognostic factor for predicting long-term survival.^3^, ^4^, ^5^, ^6^, ^7^ The response to chemotherapy has been reported to be poor in up to 40–45 % of COS.^8^^,^^9^ Neither tailoring adjuvant chemotherapy according to the histological response to neoadjuvant chemotherapy nor dose intensification has improved survival rates.^8^^,^^9^ Notably, 20–40 % of patients exhibit disease progression (DP, i.e., the development of any new lesions or increase in the size of existing lesions) while on chemotherapy, with an associated 5-year OS of 13–40 % and EFS of 6–27 %.^10^, ^11^, ^12^

Factors previously associated with disease progression include older age, axial skeleton, positive surgical margins, poor chemotherapy response, multiple site metastases, and incomplete surgical remission.^14-16.^ However, there is a paucity of data on COS disease progression in low- or middle-income countries (LMICs), particularly in Africa. It remains unclear whether treatment protocols adopted from the developed world are optimal.^4^^,^^13^^,^^12^ Identifying factors predictive of disease progression, especially while undergoing chemotherapy, has important clinical implications. If a patient can be identified as being at risk of disease progression while receiving chemotherapy, the use of neoadjuvant chemotherapy with a resulting delay in surgery may be inappropriate. In addition, novel therapies must be considered earlier to increase the probability of survival.

We aimed to determine the prognostic factors associated with disease progression in central high-grade conventional osteosarcoma of the appendicular skeleton treated at a single centre in South Africa with a minimum 3-year follow-up period.

## 2 Methods

### 2.1 Patients and study Design

**Fig. 1:**
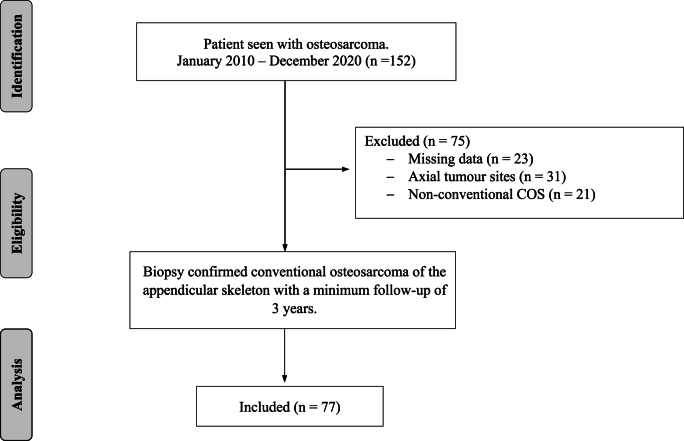
STROBE flow chart of patients with high-grade central conventional osteosarcoma of the appendicular skeleton selected for inclusion treated with adjuvant chemotherapy: Single-center within South Africa 3-year minimum follow-up.

For this retrospective study, we collected and analyzed data from our institution's electronic database over a 10-year period, with a minimum of 3-year follow-up. The study proceeded only once all the necessary ethical and local regulatory approvals were obtained. We identified 152 osteosarcoma patients seen at our training hospital between January 2010 and December 2020. We included all patients with biopsy-histologically confirmed central high-grade conventional osteosarcomas of the appendicular skeleton. Seventy-five patients met the exclusion criteria. The remaining 77 patients were included in the analysis. [Fig. 1].

The assimilated variables included comprised age, sex, duration of symptoms before the presentations (in months), days from presentation to a definitive diagnosis, tumour location (sites), presence of pathological fracture, metastasis at diagnosis i.e. Enneking stage, metastatic site, serum alkaline phosphatase (ALP), lactate dehydrogenase (LDH), serum albumin, histological subtype, the treatment strategy employed (being either curative with surgery, or palliative for unresectable tumours), Enneking wide or radical surgical excisions performed, mean resection margin), and neoadjuvant and adjuvant chemotherapy was used. The tumour volume was assessed using MRI and measured using a previously defined formula for an ellipsoidal mass (width × height × diameter × 0.52).^14^^.^

### 2.2 Outcome measures

Disease progression (DP) was defined in line with the Response Evaluation Criteria in Solid Tumours (RECIST) recommendations and the multistate model for the EURAMOS-1 study.^15^^,^^16^ In our study, we classified DP as (1) local progression, involving either enlargement of the primary tumour pre-operatively while the patient was on neoadjuvant chemotherapy or recurrence of the tumour following resection (local recurrence); (2) systemic progression with the development of new metastasis or the enlargement of existing metastasis by more than 20 %, either pre-or post-operatively and (3) mortality due to cancer progression as determined by clinical and radiological evaluations.^15^ Time to disease progression was measured from the date of presentation to the date of clinical or radiological evidence of tumour progression. Progression-free survival (PFS) was defined as the length of time between treatment and measurable worsening of the disease (disease progression).

### 2.3 Statistical data analysis

Using Stata statistical software, Release 18. College Station, TX, Stata Corp LLC), continuous variables were summarized as means and standard deviations (SDs). Categorical variables were summarized as counts and percentage frequencies, and associations were tested using the Chi-Square or Fischer's exact test. The Cox proportional bivariate and multivariate regression modelling for prognostic factors and the Kaplan-Meier for the survival analysis, with the statistical significance differences set at p < 0.05. The starting point was the time from the treatment's start to the event's time.

## 3 Results

### 3.1 Patient-related factors

At a minimum of 3-year follow-up, a total of 77 patients were included in the analysis. The median patient age was 19 years (Interquartile range - IQR 14.0–27.0; range 8–61), with nearly equal distribution between males and females (49.4 %; n = 38 vs. 50.6 %; n = 39) [Table 1]. The median duration of symptoms was five months (IQR 3–7 months; range 0–73 months), and the median duration from presentation to diagnosis was 12 days (IQR 7–15; range 0–527). The most common location was around the knee, which involved the distal femur (44.2 %; n = 34) and the proximal tibia (24.7 %; n = 19) [Table 2]. Additional tumour locations included comprised the proximal humerus (16.9 %; n = 13) and other long bones, – including the fibular, radius, and ulnar (14.3 %; n = 11). Almost half of the cohort presented with pathological fractures at the time of diagnosis (49.4 %; n = 38). The median MRI tumour volume was 456 cm3 (IQR 242–970; range 37– to 14489), and more patients presented with large tumours (75th percentile, 50.8 %; n = 33) than compared with medium (50th percentile, 23.1 %; n = 15) and small (25th percentile, 26.2 %; n = 17) tumours. Regarding histological subtypes, most tumours were osteoblastic (53.2 %; n = 41), followed by chondroblastic (32.5 %; n = 20), and fibroblastic (14.3 %; n = 11). There were similar proportions of patients presenting with increased serum ALP levels (39.4 %; n = 13), normal ALP levels level (39.4 %; n = 13), and decreased ALP levels (21.2 %; n = 7). Serum LDH levels were more commonly decreased (78.8 %; n = 26) than increased (9.1 %; n = 3) or normal (12.1 %; n = 4) levels. Serum albumin (Alb) levels were either normal (72.2 %; n = 24) or decreased (27.3 %; n = 9). Notably, there was an increased proportion of metastatic disease at presentation, i.e. Enneking stage III (57.1 %; n = 44). Lung only was commonly metastasised (75 %; n = 33) compared to multiple sites (25 %; n = 11).

**Table 1:** Patient factors associated with disease progression.

Patient factors	Overall (N = 77)	No progression (N = 19)	Progression (N = 58)	Univariate analysis		Multivariate analysis	
				HR (CI)	p-value	HR(CI)	p-value
**Age years**				1.01 (0.99–1.03)	0.218	1.04 (1.00–1.08)	<0.034∗
Median(Q1-Q3)	19.0 (14.0–27.0)	17.0(13.5–20.0)	21.5(14.3–28.8)				
n(Min-Max)	77 (4.00–61.0)	19(4.00–32.0)	58(8.00–61.0)				

**Sex**				0.97 (0.57–1.66)	0.911	0.91(0.41–2.01)	0.820
Female	39 (50.6 %)	10 (52.6 %)	29 (50.0 %)				
Male	38 (49.4 %)	9 (47.4 %)	29 (50.0 %)				

**Duration of symptoms months**				1.02 (0.99–1.05)	0.136	0.98 (0.91–1.06)	0.619
Median(Q1-Q3)	5.00 (3.00–7.00)	3.00(3.00–6.00)	5.50(3.00–8.00)				
n(Min-Max)	77 (0–73.0)	19(0–18.0)	58(0–73.0)				

**Time from presentation to diagnosis**				1.00 (1.00–1.01)	0.283	1.01 (1.00–1.02)	0.161
Median(Q1-Q3)	12.0 (7.00–15.0)	10.0(6.50–17.5)	12.0(9.25–15.0)				
n(Min-Max)	77 (0–527)	19(0–90.0)	58(0–527)				

**Serum albumin group**				1.25 (0.72–2.17)	0.436	1.89 (0.82–4.34)	0.132
Normal	24 (72.7 %)	11 (57.9 %)	36 (63.2 %)				
Decreased	9 (27.3 %)	8 (42.1 %)	21 (36.8 %)

**Table 2:** Tumour-related factors associated with disease progression.

Tumour-related factors	Overall (N = 77)	No progression (N = 19)	Progression (N = 58)	Univariate analysis		Multivariate analysis	
				HR (CI)	p-value	HR(CI)	p-value
**Tumour location**				2.48 (1.02–6.04)	0.046∗	2.28 (0.68–7.60)	0.180
Proximal tibia	19 (24.7 %)	3 (15.8 %)	16 (27.6 %)				
Distal femur	34 (44.2 %)	9 (47.4 %)	25 (43.1 %)				
Proximal humerus	13 (16.9 %)	4 (21.1 %)	9 (15.5 %)				
Other	11 (14.3 %)	3 (15.8 %)	8 (13.8 %)				

**Metastasis present at diagnosis**				1.91 (1.10–3.32)	0.022∗	1.16 (0.16–8.26)	0.881
No	33 (42.9 %)	22 (66.7 %)	11 (25.0 %)				
Yes	44 (57.1 %)	11 (33.3 %)	33 (75.0 %)				

**Site of metastasis**				2.58 (1.13–5.88)	0.024∗	–	–
Lung only	33 (75.0 %)	8 (42.1 %)	25 (43.1 %)				
Multiple	11 (25.0 %)	11 (57.9 %)	33 (56.9 %)				

**MRI volume group**				1.00 (0.38–1.67)	0.141	–	–
Small	17 (26.2 %)	11 (37.9 %)	6 (16.7 %)				
Medium	15 (23.1 %)	5 (17.2 %)	10 (27.8 %)				
Large	33 (50.8 %)	13 (44.8 %)	20 (55.6 %)				

**Histological subtype**				2.3 (0.29–1.92)	0.936	–	–
Osteoblastic	41 (53.2 %)	18 (54.5 %)	23 (52.3 %)				
Chondroblastic	25 (32.5 %)	10 (30.3 %)	15 (34.1 %)				
Fibroblastic	11 (14.3 %)	5 (15.2 %)	6 (13.6 %)				

**Serum alkaline phosphatase**				1.17 (0.52–2.63)	0.713	0.47(0.12–1.86)	0.282
Normal	13 (39.4 %)	4 (21.1 %)	17 (29.3 %)				
Increased	13 (39.4 %)	5 (26.3 %)	10 (17.2 %)				
Decreased	7 (21.2 %)	10 (52.6 %)	31 (53.4 %)				

**Serum lactate dehydrogenase**				1.17 (0.56–2.43)	0.675	1.96 (0.68–5.65)	0.211
Normal	4 (12.1 %)	1 (5.3 %)	10 (17.5 %)				
Decreased	26 (78.8 %)	13 (68.4 %)	37 (64.9 %)				
Increased	3 (9.1 %)	5 (26.3 %)	10 (17.5 %)				
							

### 3.2 Treatment-related factors

Thirty-two per cent (n = 25) of the patients underwent curative treatment, involving wide resection of all sites and adjuvant chemotherapy [Table 3]. Sixty-eight per cent (n = 52) of the patients were treated palliatively, where resections of all sites were deemed impossible. Enneking wide or radical surgical excision of the primary tumor performed (68 %; n = 52), irrespective of the treatment approach (curative or palliative). In the same vein, radical limb ablation was commonly performed in patients who underwent surgical management (58 %; n = 45). Of these, 80 % (n = 62) received upfront surgery (immediate) without pre-operative chemotherapy, and only 20 % (n = 15) received neoadjuvant chemotherapy. Conversely, 75 % of patients (n = 58) received adjuvant chemotherapy. Finally, 56 % (n = 43) of the patients exhibited mean surgical resection margins of >10 mm.

**Table 3:** Treatment factors associated with disease progression.

Treatment factors	Overall (N = 77)	No progression (N = 19)	Progression (N = 58)	Univariate analysis		Multivariate analysis	
				HR (CI)	p-value	HR(CI)	p-value
**Curative**				0.33 (0.17–0.62)	<0.001∗	0.38 (0.09–1.65)	0.198
No	52 (67.5 %)	12 (63.2 %)	40 (69.0 %)				
Yes	25 (32.5 %)	7 (36.8 %)	18 (31.0 %)				
**Palliative**				0.33 (0.17–0.62-)	<0.001∗	0.38 (0.09–1.65-)	0.198
No	25 (32.5 %)	7 (36.8 %)	18 (31.0 %)				
Yes	52 (67.5 %)	12 (63.2 %)	40 (69.0 %)				
**Wide or radical surgical excisions**				0.48 (0.27–0.86)	<0.013∗	1.69 (0.33–8.55)	0.528
No	25 (32.5 %)	7 (36.8 %)	18 (31.0 %)				
Yes	52 (67.5 %)	12 (63.2 %)	40 (69.0 %)				
**Limb ablation**				0.73 (0.42–1.25)	0.251	0.57 (0.04–7.82)	0.674
No	32 (41.6 %)	8 (42.1 %)	24 (41.4 %)				
Yes	45 (58.4 %)	11 (57.9 %)	34 (58.6 %)				
**Upfront surgery**				1.13 (0.59–2.14)	0.717	1.38 (0.30–6.36)	0.678
No	15 (19.5 %)	1 (5.3 %%)	14 (24.1 %)				
Yes	62 (80.5 %)	18 (94.7 %%)	44 (75.9 %)				
**Mean resection margins(10 mm)**				1.71 (0.99–2.93)	0.052	2.27 (0.72–7.16)	0.162
>10 mm	43 (55.8 %)	11 (57.9 %)	32 (55.2 %)				
<10 mm	34 (44.2 %)	8 (42.1 %)	26 (44.8 %)				
**Neoadjuvant chemotherapy**				1.13 (0.59–2.14)	0.717	1.38 (0.30–6.36)	0.678
No	62 (80.5 %)	18 (94.7 %)	44 (75.9 %)				
Yes	15 (19.5 %)	1 (5.3 %)	14 (24.1 %)				
**Adjuvant chemotherapy**				0.36 (0.09–1.38)	0.127	0.39 (0.14–1.06)	0.066
No	19 (24.7 %)	7 (36.8 %)	12 (20.7 %)				
Yes	58 (75.3 %)	12 (63.2 %)	46 (79.3 %)				

### 3.3 Disease progression patterns (DPs)

Seventy-five per cent of the patients exhibited disease progression (58/77), and of these, 76.1 % (44/58) demised within 3 years. Thirty-five per cent (27/77) of patients had new lesions or new lesions/metastasis. The DPs patterns were either local in 32.5 % (25/77), systemic in 61 % (47/77), or combined in 31.2 % (24/77). The sites of DPs comprised lung 19.5 % (15/77), bone 11.7 % (9/77), brain 1.3 % (1/77), and liver 1.3 % (1/77), respectively. DP while on neoadjuvant chemotherapy 18.2 % (14/77). In contrast, 55.9 % (43/77) exhibited DP on adjuvant chemotherapy. Local progressions were treated using either Enneking wide/radical surgical resections 16.9 % (13/77) or radiation 12.9 % (10/77). Systemic progressions were treated either surgically with metastasectomies 6.5 % (5/77) or salvage chemotherapy - ifosfamide 19.5 %, (9/77), etoposide 11.7 % (9/77); gemcitabine 11.7 %, (9/77), docetaxel 11.7 %, (9/77), and cyclophosphamide 3.4 %, (2/77). Nineteen – per cent (15/77) of the patients received lung radiation therapy.

### 3.4 Factors associated with disease progression and survival

In the univariate analysis, poor prognostic factors associated with DP were proximal humerus tumour site (Hazard ratio [HR] 2.48; confidence intervals [CI] 1.02 to 6.04; p < 0.046); metastasis at diagnosis i.e. Enneking stage III (HR 1.91; CI 1.10 to 3.32; p < 0.022), multiple metastatic lesions (HR 2.58; CI 1.13 to 5.88; p < 0.024), and palliative treatment (HR 2.17; CI 1.24 to 3.78; p < 0.007). In contrast, the protective factors were comprised of curative treatment (HR 0.33; CI, 0.17 to 0.62; p < 0.001) and wide/radical resections (HR 0.48; CI, 0.27 to 0.86; p < 0.013). None of the other factors assessed were associated with disease progression, including the resection margins, amputation, and upfront surgery without neoadjuvant and adjuvant chemotherapy [Table 3]*.*

**Fig. 2:**
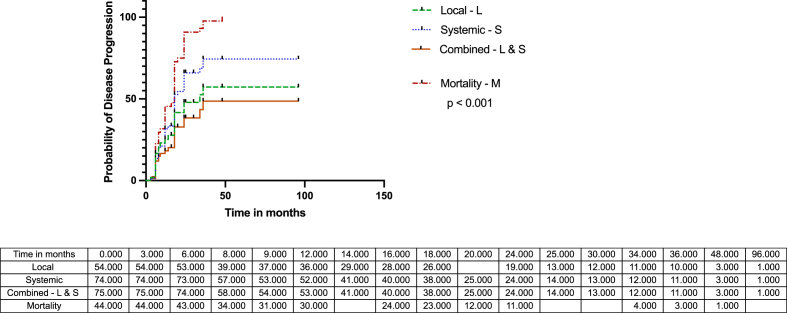
The accumulative incidences of central high-grade conventional osteosarcoma of the appendicular skele median time to progression-free survival (PFS) were 34 and 18 months for local and systemic progression, respectively, and 18 months for mortality (p < 0.001)

**Fig. 3:**
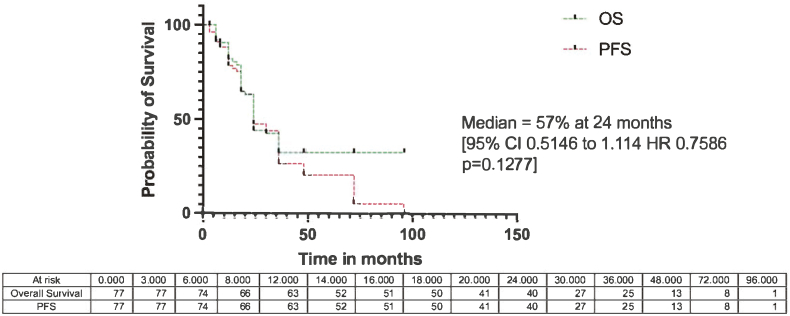
The Kaplan Meier overall and progression-free survival of central high-grade conventional osteosarcoma with a South African single centre.

In the multivariate regression analysis, only increasing age emerged as a significant patient-related predictor of disease progression (HR 1.04; CI 1.00 to 1.08; p < 0.034) [Table 1, Table 2]*.* The multivariate analysis did not predict all treatment factors, including the curative treatment strategy, wide/radical resections, amputations, resection margins, neoadjuvant chemotherapy, and adjuvant chemotherapy [Table 3]. The median time to progression-free survival (PFS) were 34 and 18 months for local and systemic progression, respectively, and 18 months for mortality (p < 0.001) [Fig. 2]. The median survival was 24 months, with a projected 5-year Kaplan- Meier overall survival of 29.8 % and PFS of 10.3 % (HR 0.76; CI 0.5146 to 1.114; p < 0.128), respectively. [Fig. 3].

## 4 Discussion

While our cohort exhibited clinical characteristics similar to those reported globally,^2^, ^3^, ^4^, ^5^, ^6^, ^7^, ^13^, ^17^, ^18^, ^19^ but a discordance was observed with disease progression (DP) and survival outcomes.^10^, ^11^, ^12^ In line with global reports,^14^ ^– 16, 19-24^ we found that poor prognostic factors for DP included increasing age, proximal humerus tumor location, metastasis at diagnosis, multiple metastases, and palliative treatment. In contrast, wide surgical resection and curative treatments were beneficial. Albeit the effect size was small, increased age was an independent predictor. However, this aligns with North America and Africa, with a decreased likelihood of survival after relapse with increasing age.^20^^,^^21^^,^^22^ The hypothetical plausible explanation could be variations in tumor biology and/or chemotherapy intolerances with the increasing age.

Our progressed osteosarcoma exhibited a median overall survival (OS) of 24 months with 44 events (mortality) and a projected 5-year OS of 30 % and progression-free -survival probability (PFS) of 10 %, respectively. Our results are in keeping with impoverished survival outcomes of osteosarcoma relapse reported in high-income countries (HICs) at < 30 % at five years and EFS at < 40 %.^14 – 15, 21 – 22, 24^ Spraker-Perlman's et al. largest prospective cohort of newly diagnosed osteosarcomas (n = 777) from 3 North American trials and found a 5-year survival rate of only 17 % after a first recurrence.^20^ However, in our series, the absence of a larger sample size, a national osteosarcoma database, and standardised national guidelines for refractory osteosarcomas underscores the need for further research in this area.

While the global DP rate ranges from 8 % to 35 %, we observed a unique higher proportion of DP at 75 %. ^14-16.^ However, our high metastatic rate, i.e. Enneking stage III, at 57 % must be considered. Sixty-one per cent of COS exhibited systemic progression (SP) with a median time to an event at 18 months, manifesting as metastases to the lung, bone, liver, and brain. In HICs, the advanced disease is less commonly observed compared to South Africa, where metastases have been reported to be present in 47%–80 % of patients on presentation,^13^, ^17^, ^18^, ^19^ in contrast with global figures typically below 20 %.^3^, ^4^, ^5^, ^6^, ^7^ This regional discrepancy begs further elucidation. Whether this is related to tumour biology, delayed clinical presentation, or healthcare access disparities remains unclear. In our series, patients with metastasis had a two-fold increase in the likelihood of progression. Lung metastasis was associated with a three-fold increase in the risk of developing DP. In previous studies, multiple pulmonary metastases were also indicative of poorer prognosis for DP and OS in osteosarcoma.^20^ In line with HICs,^10^^,^^23^ SP was treated surgically with metastasectomy, salvage chemotherapy, and radiation. While there is a general consensus about the effectiveness of surgical resections of all sites in DP, the use of salvage chemotherapy or dose intensification has been questioned.^8^, ^9^, ^10^, ^11^, ^12^ Albeit micrometastasis has slowed, it does not impart any survival benefit.^8^^,^^9^ Despite the use of salvage chemotherapy in our clinical practice, 35 % developed new lesions during or after treatment. In resource-constrained environments like ours, there are very few alternative treatment options for chemo-resistant COS phenotypes.

Thirty-two per cent of COS exhibited local progression (LP), with a median time to an event of 34 months. LP while on neoadjuvant chemotherapy was 18.5 %. DP in osteosarcoma before local control confers a poor prognosis.^24^ Again, routine use of neoadjuvant chemotherapy has also been questioned.^24^ Although it may facilitate limb-salvage surgery, it does not impart a survival benefit over upfront surgery.^25^ Similarly, in our series, neither neoadjuvant chemotherapy nor upfront surgery yielded survival benefits. This aligns with the Paediatric Oncology Group that previously found comparable event-free survival (EFS) rates for immediate surgery and presurgical chemotherapy (69 ± 8 % vs. 61 ± 8 %).^28.^ In keeping with HICs,^11^^,^^24^^,^^26^ our LP of osteosarcomas was treated using either Enneking wide/radical surgical resections 16.9 % or local radiation 12.9 %, respectively.

One-third of our patients were treated curatively, with wide resection of all sites and chemotherapy (MAP), and 31 % of these progressed. The curative approach significantly decreased the likelihood of DPs by 33 %. The curative approach is considered to be the gold standard for acceptable outcomes in COS.^4^, ^5^, ^6^, ^7^, ^13^, ^17^, ^18^, ^19^^,^^10^ Thebault et al. achieved a 3-year PFS and OS rates of 21 % and 37 %, respectively, with a curative approach.^10^ In contrast, multiple relapses in COS pose a significant challenge; the unanswered question is when to stop the aggressive treatments in cases with DP? We performed Enneking wide/radical resection surgery for primary tumours in 68 %, reducing the likelihood of DP by nearly 50 %. Wide surgical resection of all sites, with tumour-free surgical margins, in COS, yielded better outcomes.^15, 29 – 30^ A high proportion of our osteosarcomas (55 %) were treated palliatively amid surgical irresectability, and 57 % of these cases exhibited DP with a 2-fold increase in likelihood. Palliatively treated COS are less likely to survive in our clinical practice.^13^

There are numerous limitations to this study. The small sample size is a significant shortcoming, and our findings should be interpreted cautiously. However, a post hoc power analysis suggested that our study may be adequately powered regarding the primary outcome of DP. The retrospective nature of the data collection meant that we could only analyze some possible factors that may have a bearing on the prognosis. Several patients were lost to follow-up, precluding longer-term longitudinal prognostication. Loss to follow-up is due to refusal of treatment (amputation or chemo-intolerances), non-Western medicines, and socio-economic factors. In Africans, there is an increase in incidences, unknown risk factors, and a high propensity for metastatic disease for COS. ^8-11, 31.^

## 5 Conclusion

In this series of central high-grade conventional osteosarcoma of the appendicular skeleton from South Africa, we observed a uniquely high proportion of disease progression (DP). Age >19, metastatic disease, and no chemotherapy response yielded impoverished outcomes; in contrast, wide surgical resection is beneficial. Further elucidation is needed at the larger scale in this region.

## CRediT authorship contribution statement

**PhakamaniG. Mthethwa:** Conceptualization, Formal analysis, Investigation, Methodology, Project administration, Writing – original draft. **L.C. Marais:** Conceptualization, Methodology, Writing – original draft, C.M. Aldous: Conceptualization, supervision, review and editing.

## Data Sharing

The datasets generated and analyzed in the current study are not publicly available due to data protection regulations. Access to data is limited to the researchers who have obtained permission for data processing. Further inquiries can be made to the corresponding author.

## Funding statement

The authors received no financial or material support for the research, authorship, and/or publication of this article.

## Icmje COI statement

The authors declare no conflicts of interest. All authors have read and agreed to the published version of the manuscript.
